# Remission of chronic actinic dermatitis on baricitinib: A case report

**DOI:** 10.1002/ski2.243

**Published:** 2023-11-08

**Authors:** Jessica Maguire, David Gleeson, Roberto Corso, Andrew Pink, Catherine Smith, John Ferguson

**Affiliations:** ^1^ St John's Institute of Dermatology Guy's and St Thomas' NHS Foundation Trust London UK; ^2^ St John's Institute of Dermatology Kings College London and Guy's and St Thomas' NHS Foundation Trust London UK

## Abstract

Chronic actinic dermatitis (CAD) is an immune‐mediated photodermatosis characterised by eczematous, pruritic changes to sun‐exposed skin. The pathophysiology of CAD is poorly understood, with current explanations including a hypersensitivity reaction and cross‐reactivity to contact allergens. The disease is often refractory to immunosuppressive treatment and has a marked impact on patient quality of life. Janus kinase inhibitors (JAKi) are a novel class of small molecules licenced for the management of certain inflammatory conditions, including atopic dermatitis We present the case of a 69‐year‐old gentleman with a history of severe CAD, unresponsive to standard therapies, who was prescribed baricitinib, a janus kinase (JAK) inhibitor as a single agent treatment for his disease. The patient experienced a dramatic clinical improvement with this therapy. In addition, normalisation of photo test and improvement of patch test results following treatment were observed. There is one previous case report in the literature describing the clinical response of patients with CAD to JAK inhibitor therapy, but no comment on pre or post treatment photo testing, patch testing or photo‐patch testing results was made. In this case report, we discuss our understanding of the role of JAK inhibitors in CAD and highlight a potential new therapeutic avenue for this disabling disease.

## Graphical abstract

Chronic actinic dermatitis (CAD) is characterised by eczematous, pruritic changes to sun‐exposed skin. It is often refractory to treatment, with a marked impact on patient quality of life. We present the case of a 69 year old gentleman with severe CAD, who has achieved complete clinical and photo‐test remission of his disease following treatment with the janus kinase (JAK) inhibitor baricitinib.

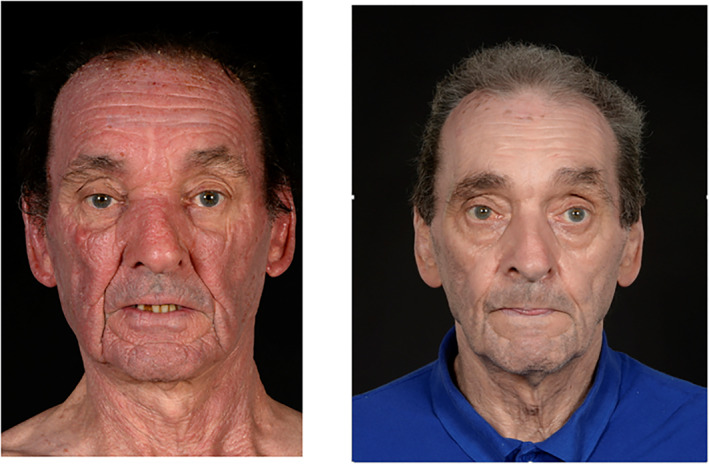

## 1 INTRODUCTION

Chronic actinic dermatitis (CAD) is an immune‐mediated photodermatosis characterised by pruritic, eczematous lesions affecting sun‐exposed skin.^1^ Its pathophysiology is poorly understood. Current explanations favour a hypersensitivity reaction to UV‐induced neoantigens in the skin.^2^ Approximately 50% of cases report concurrent allergic contact dermatitis, suggesting cross‐reactivity to exogenous contact antigens.^3^ CAD is often refractory to immunosuppressive treatment. Janus kinase inhibitors (JAKi) are a novel class of small molecules licenced for the management of certain inflammatory conditions, including atopic dermatitis. One case report describes the clinical response of three patients with CAD to the JAK inhibitor tofacitinib^4^ but did not include pre or post treatment photo testing, patch testing or photo‐patch testing results. We present a case of treatment refractory CAD which has dramatically improved clinically, with normalisation of photo test and improvement of patch test results following treatment with baracitinib, a JAK 1,2 inhibitor.

## 2 CASE

A 69‐year‐old gentleman was referred to the specialist photobiology centre in 2005 with a 5‐year history of a photo‐distributed rash. The patient first noticed erythema and pruritus of the palms and dorsal aspect of the forearms after commencing work with Royal Mail. His rash spread to affect predominantly sun‐exposed sites, later spreading to covered sites. His symptoms worsened in summer after a few minutes in the sun. He had no prior dermatological history. He was otherwise fit and well, with no history of atopy or notable family history.

Monochromator and solar simulator testing in 2010 showed abnormal responses consistent with chronic actinic dermatitis. Patch testing in 2013 showed allergic reactions to cobalt, thiuram and epoxy resins. In 2014, reactions were also noted to methylisothiazolinone, colophony and clobetasol. In 2017, photo‐patch tests were positive to colophony, epoxy resin, evernia furfuracea and linalool. Despite stringent allergen avoidance, symptoms persisted.

Initial treatment comprised potent topical corticosteroids and multiple weaning courses of systemic steroid. Systemic therapy was commenced in 2012 with azathioprine 50 mg daily but stopped after nine days due to intolerable gastrointestinal side effects. Over the following 9 years, multiple other systemic agents were trialled including mycophenolate mofetil, methotrexate, acitretin and alitretinoin. Each demonstrated limited therapeutic effect, requiring adjuvant therapy with further courses of topical and systemic steroid. The patient's quality of life deteriorated, remaining indoors during summer and winter months. In June 2021, baricitinib 2 mg orally once daily was commenced as single‐agent treatment for CAD. Baseline disease severity scores were markedly raised, with DLQI 25, PHQ9 22, GAD7 14 and POEM 28.

At week seven of treatment the patient developed blistering on the tips of his fingers. This resolved with flucloxacillin and empiric valaciclovir. Baricitinib was restarted at week 9 at an increased dose of 4 mg daily. At 9 months, the patient reported improvement, with an EASI score of 0.5 and DLQI 2 (a 90% improvement in his scores). At 16 months, he had clear skin and could go gardening on sunny days without sun protection. By November 2022 repeat photo testing was normal. Repeat patch testing showed persistent allergy to colophony, epoxy resin and clobetasol. Photo‐patch testing showed a new reaction to diclofenac, notably only apparent on the patch exposed to UVA at 5 J/cm^2^, higher than the patient ‘s previous exposure, given his disease severity.

## 3 DISCUSSION

**FIGURE 1:**
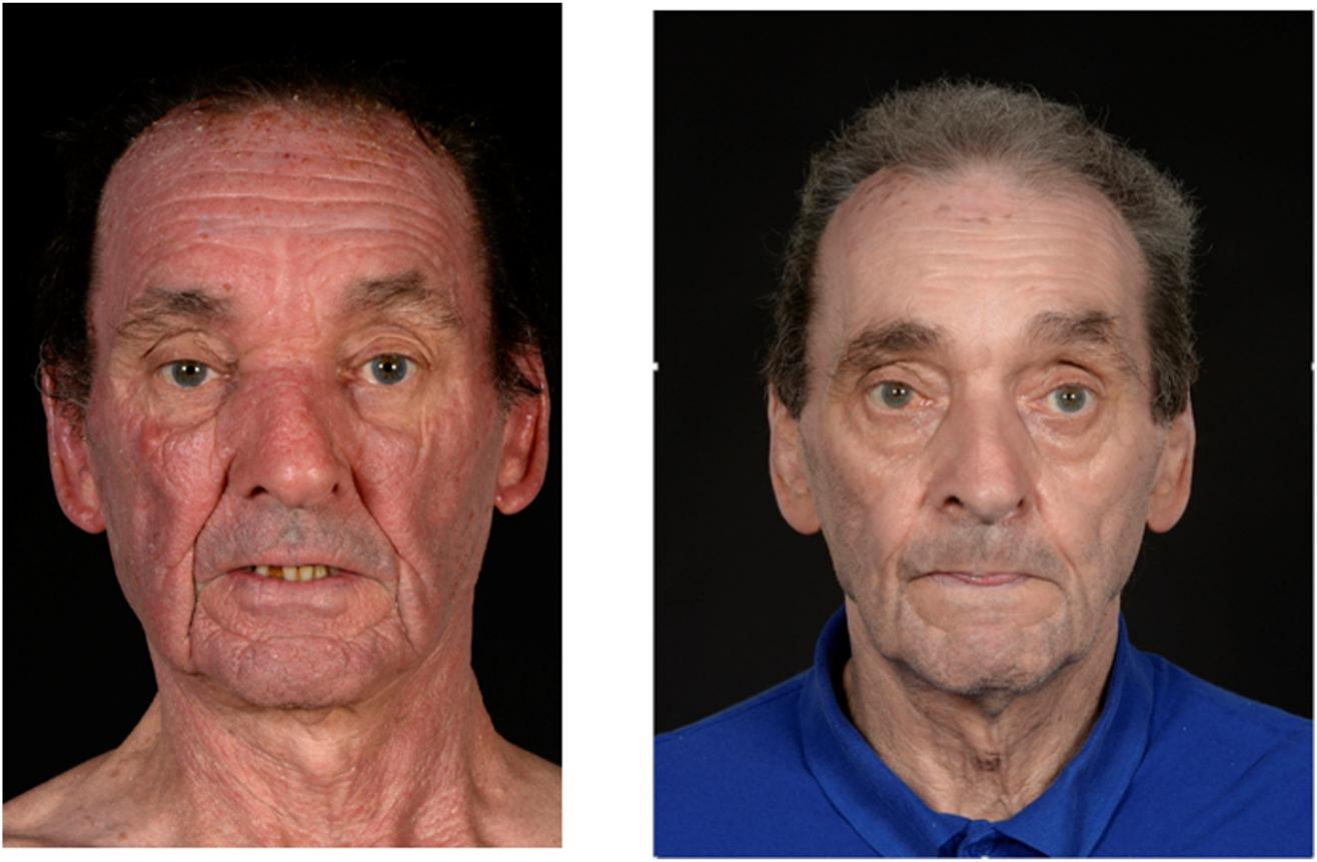
Frontal view of patient depicting improvement in skin post commencement of baricitinib.

**FIGURE 2:**
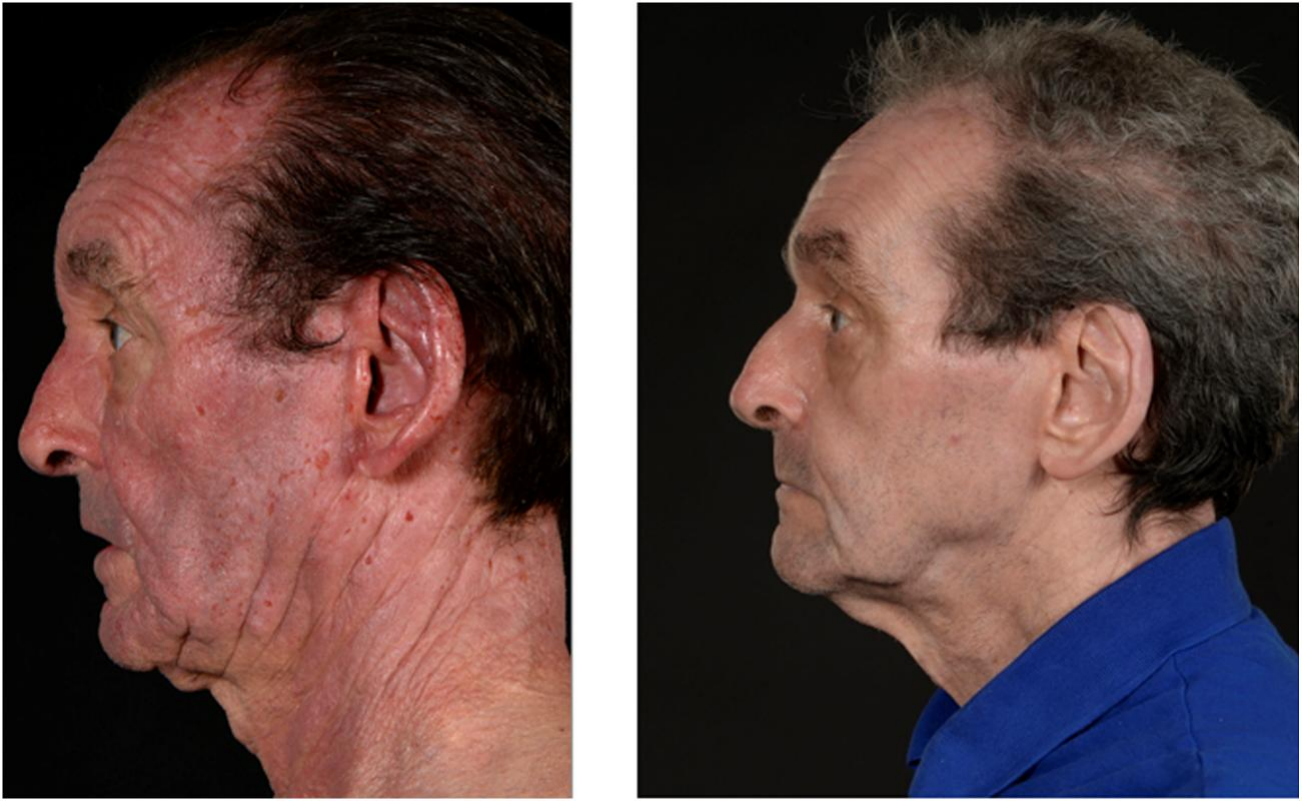
Profile view of patient depicting improvement in skin post commencement of baricitinib.

**FIGURE 3:**
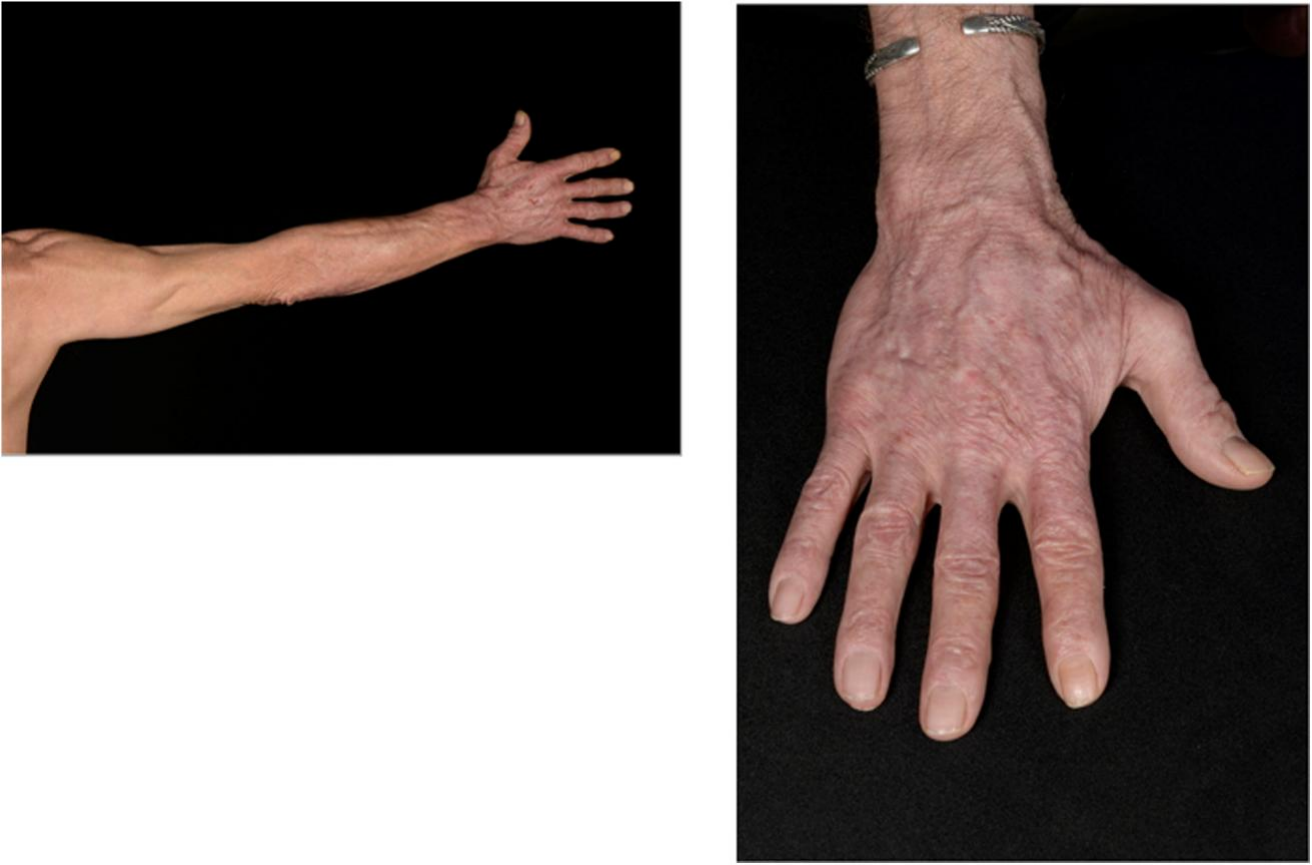
View of right dorsal forearm and hand depicting improvement in skin post commencement of baricitinib.

To our knowledge, this is the first case of CAD reported in the literature to respond to JAK inhibitor therapy as single‐agent treatment (as illustrated in Figures 1, 2, 3), with induction of complete clinical remission, and normalisation of photo‐testing. CAD was first described by Ian Magnus in 1969 in a case series of 10 patients at the St John's Institute of Dermatology with persistent photo‐sensitive dermatitis.^5^ Consistent with our case, CAD is characterised by eczematous, pruritic, and often lichenified changes to sun‐exposed skin. The face, scalp, neck, upper chest, and dorsal aspect of hands and forearms are the worst affected sites.^6^

Patients of all ages and ethnicities can be affected.^7^ Important investigations include photo testing, patch testing and photo‐patch testing. Photosensitivity to UV wavelengths, particularly UVB but also UVA, and sometimes visible wavelengths, is described.^6^ Lupus serology is usually negative.^8^ Treatment includes strict sun avoidance, use of sunscreens, emollients, and topical corticosteroids. Standard systemic immunosuppression is considered if CAD is not controlled with skin‐directed measures. The pathophysiology of CAD is poorly understood. However, given its clinical and histopathological appearances and the presence of CD8 positive T cells in the dermis, a hypersensitivity reaction akin to persistent allergic contact dermatitis is broadly accepted to be the mechanism^8^; causative allergens are endogenous and photo‐induced. Most cases exhibit pre‐existing allergic contact dermatitis.^3^ It has been suggested that UV‐damaged DNA may trigger an allergic contact response which, sometimes, is due to contact‐dermatitis‐enhanced immune reactivity.^8^

JAKi are a novel class of oral immunosuppressant medications licenced for the management of atopic dermatitis, psoriatic, and rheumatoid arthritis and alopecia areata. By inhibiting the action of four key tyrosine kinases, JAK1, JAK2, JAK3 and tyrosine kinase 2 (TYK2), they affect various cell lineages including CD8 positive T cells, and suppress interferon gamma and interleukin pathways. Baricitinib is a JAK 1,2 inhibitor, used to treat atopic eczema. Given current evidence suggesting CAD as a cytotoxic T cell mediated disease, one could infer a therapeutic role for JAK pathway inhibition. Our patient's dramatic response to treatment with baricitinib supports this hypothesis.

**FIGURE 4:**
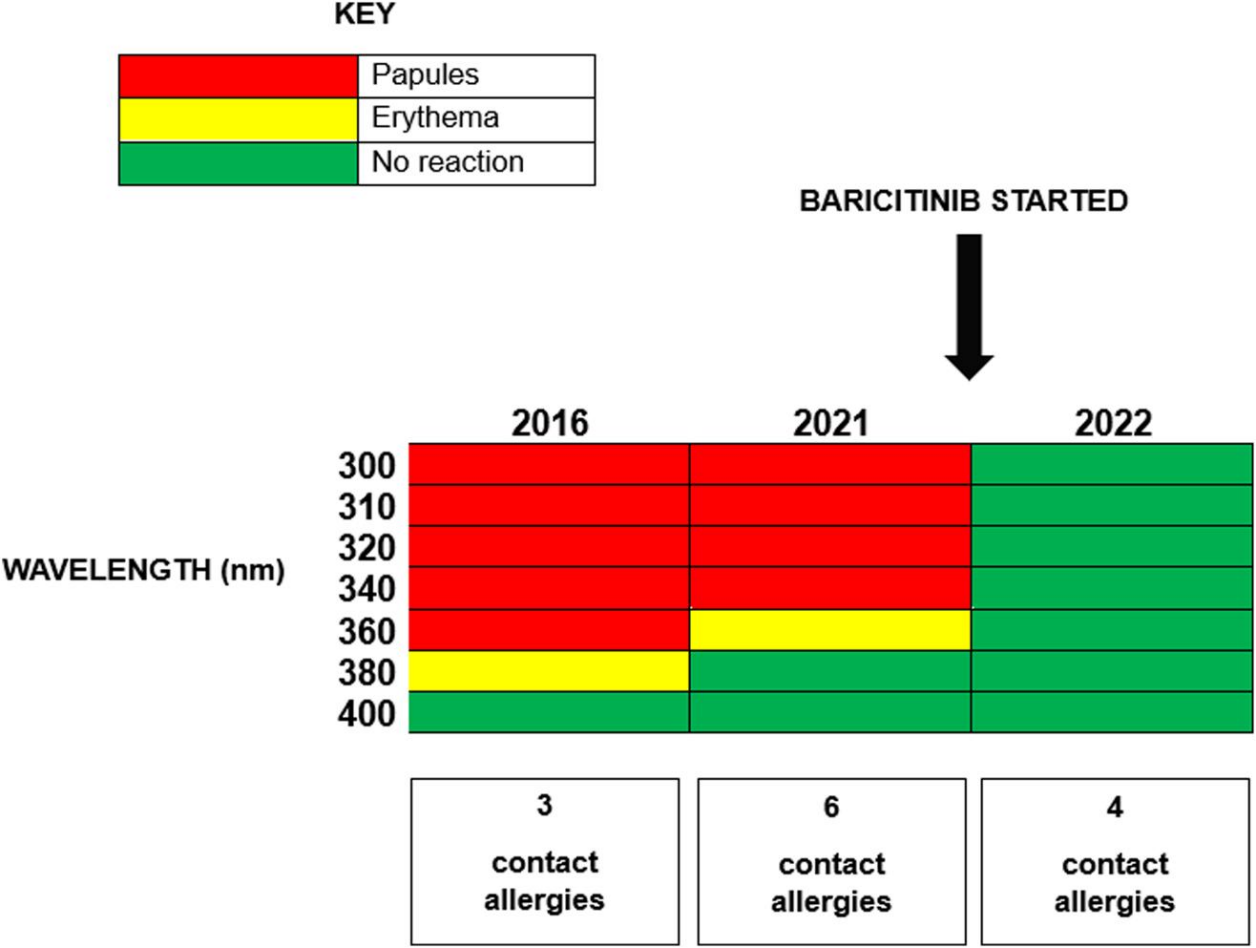
Monochromator testing results by year and wavelength, with associated number of contact allergies.

Spontaneous remission of CAD has been described by Dawe et al. One in five patient's abnormal photosensitivity resolves 10 years after diagnosis.^3^ However, the presence of multiple contact allergens in two or more patch test series, as in our patient's case, is usually a predictor of poor prognosis for spontaneous resolution of disease.^3^ As shown in Figure 4, normalisation of monochromator testing in the presence of multiple contact allergens suggests that the improvement in our case may be attributed to baricitinib, rather than spontaneous resolution. We propose that treatment be paused following one clear summer period, with regular follow up to monitor for recurrence of skin disease.

## 4 CONCLUSION

Our patient with treatment refractory CAD has achieved full clinical remission of disease and normalisation of photo test results with baricitinib treatment. We propose that JAK pathway inhibition represents a potential new therapeutic avenue for this disabling disease and that consideration be given to baricitinib earlier in the treatment pathway.

## CONFLICT OF INTEREST STATEMENT

Professor Catherine Smith is an Investigator on EU‐IMI funded consortium with multiple industry partners (see biomap‐imi. eu and hippocrates‐imi. eu) and receives departmental research funding from Boehringer Ingleheim. Dr John Ferguson is a paid advisor for Incyte Pharmaceuticals and Chief Investigator for Pfizer, examining the use of ritlecitinib in vitiligo.

## AUTHOR CONTRIBUTIONS

**Jessica Rachel Maguire**: Conceptualization (equal); Data curation (lead); Project administration (lead); Writing – original draft (lead); Writing – review & editing (lead).  **David Gleeson**: Conceptualization (equal); Project administration (supporting); Writing – original draft (supporting); Writing – review & editing (supporting). **Roberto Corso**: Conceptualization (supporting); Project administration (supporting); Writing – review & editing (supporting). **Andrew Pink**: Conceptualization (supporting); Supervision (supporting). **Catherine Smith**: Conceptualization (equal); Supervision (equal); Writing – review & editing (equal). **John ferguson**: Conceptualization (equal), Supervision (equal); Writing – review & editing (equal).

## ETHICS STATEMENT

Not applicable.

## Data Availability

Data sharing is not applicable to this article as no new data were created or analyzed in this study.
